# Correlation Analysis between Microbial Communities and Flavor Compounds during the Post-Ripening Fermentation of Traditional Chili Bean Paste

**DOI:** 10.3390/foods13081209

**Published:** 2024-04-16

**Authors:** Quanye Wu, Zhaona Xu, Shirong Feng, Xunzhu Shi, Likang Qin, Haiying Zeng

**Affiliations:** 1 School of Liquor and Food Engineering, Guizhou University, Guiyang 550025, China; 18275511056@139.com (Q.W.); 13985636914@163.com (Z.X.); lkqin@gzu.edu.cn (L.Q.); 2 Sichuan Gulin Langjiu Distillery (Luzhou) Co., Ltd., Luzhou 646601, China; 3 Zunyi Zhongyuanyuan Food Co., Zunyi 563125, China; 18685640626@139.com; 4 Majiang Mingyang Food Co., Majiang 557600, China; 18785505739@139.com

**Keywords:** chili bean paste, microbial community, volatile flavor compound, correlation analysis

## Abstract

Chili bean paste is a traditional flavor sauce, and its flavor compounds are closely related to its microflora. This study focused on investigating the content of bioactive compounds, flavor compounds, and microbial communities during the post-ripening fermentation of chili bean paste, aiming to provide a reference for improving the flavor of chili bean paste by regulating microorganisms. Compared to no post-ripening fermentation, the content of organic acids increased significantly (*p* < 0.05), especially that of citric acid (1.51 times). Glutamic acid (Glu) was the most abundant of the 17 free amino acids at 4.0 mg/g. The aroma profiles of the samples were significantly influenced by fifteen of the analyzed volatile compounds, especially methyl salicylate, methyl caproate, and 2−octanol (ROAV > 1). *Latilactobacillus* (27.45%) and *Pseudomonas* (9.01%) were the dominant bacterial genera, and *Starmerella* (32.95%) and *Pichia* (17.01%) were the dominant fungal genera. *Weissella*, *Lacticaseibacillus*, *Pichia*, and *Kazachstania* had positive effects on volatile flavoring compounds, which enriched the texture and flavor of the chili bean paste. Therefore, the microbial-community activity during the post-ripening fermentation is the key to enhance the flavor quality of the product.

## 1. Introduction

Chili bean paste, a traditional condiment fermented from red chilies, broad beans, and flour for 3–8 months [1], is widely used as the main ingredient in Sichuan, Guizhou, and Hunan cuisine for its rich aroma and fresh and spicy taste [2]. The production of traditional chili bean paste is an open solid-state fermentation process involving the association of various microorganisms with flavor compounds. Further, the formation of flavor compounds in chili bean paste occurs in two main stages: pre-fermentation and post-ripening fermentation [3]. The pre-fermentation stage refers to the koji-making stage of broad beans and the pretreatment stage of the chili sauce, while the post-ripening fermentation stage is primarily the maturation process during which the broad bean curd and chili sauce are mixed at the appropriate proportion and then placed into an open fermentation jar to continue fermenting for several months. The post-ripening fermentation stage is the key stage for the formation of flavor substances. During the post-ripening fermentation process, the large molecules of the raw materials are broken down into peptides, amino acids, and various volatile flavor substances by microorganisms [4]. Li et al. [5] discovered that the primary volatile flavor compounds in chili bean paste were alcohols, esters, and aldehydes. Previous studies have shown that *Lactobacillus*, *Bacillus*, *Enterobacter*, and *Pichia* are the core microorganisms in bean paste [4,6]. Research on the relationship between microorganisms and flavor compounds in bean paste showed that *Lactobacillus*, *Aspergillus*, and *Bacillus* positively influenced certain flavor compounds (such as Asp, Leu, and 2-acetyl pyrrole) [7]. However, differences in the variety and origin of raw materials, as well as different fermentation processes, have effects on the microbial communities and flavor characteristics of chili bean paste.

Chili bean paste is also a well-known traditional spice in Guizhou, but its manufacturing process differs from that made in Sichuan. Guizhou chili bean paste is fermented without flour, using only chili and broad beans. This reduces the problem of the paste sticking to the pan during cooking, especially long-cooked ingredients such as those in hot pots. At present, there are few reports on preparing flourless raw fermented chili bean paste in Guizhou, and none on the relationship between microorganisms and flavor compounds during the post-ripening fermentation of flourless raw fermented chili bean paste.

Therefore, the present study explores changes in the flavor compounds, the succession of microbial communities, and the correlation between microorganisms and flavor compounds during the post-ripening fermentation of flourless chili bean paste. The study’s findings offer a theoretical basis for future improvements to product flavor and the development of new products through microbial regulation.

## 2. Materials and Methods

### 2.1. Samples and Chemicals

Chili bean paste was provided by Zunyi Zhongyuanyuan Food Co., Ltd. (Zunyi, China), which was representative of chili bean paste enterprises in Guizhou Province. For this study, samples from two groups, post-fermentation at 0 months (BP0) and post-fermentation at 6 months (BP6), were collected in triplicate from the fermentation cylinder after the spontaneous fermentation phase. All chemicals were purchased from Sigma (Shanghai, China) unless otherwise noted.

### 2.2. Determination of Basic Nutritional Indicators

The sensory evaluation was performed using the geographical indication of Pixian broad bean paste [8]. The moisture content was determined according to the National Food Safety Standard [9]. Salinity was determined using the silver nitrate titration method [10]. The protein content was determined using a Kjeldahl nitrogen tester (K1100, Hanon Advanced Technology Group Co., Ltd., Jinan, China). The fat content was determined with reference to the National Standard Method for Food Safety [11]. The pH was determined using a pH meter (PHS-3C, Shanghai, China). The total acids and amino acid nitrogen were determined according to the method of Zhang et al. [12]. The reducing sugar was analyzed using the 3,5-dinitrosalicylic acid (DNS) method [13]. 

### 2.3. Determination of Amino Acids

Free amino acids were analyzed using an automated amino acid analyzer (S-433D, Sykam, Eresing Germany) according to the method of Tian et al. [14].

### 2.4. Determination of Organic Acids

A 2.5 g sample was extracted with 25 mL of ultra-pure water in a water bath at 75 °C for 20 min. The sample was cooled to room temperature in a 50 mL volumetric flask and filtered. After centrifuging the filtrate for 20 min at 4000 rpm, a 0.45 μm microporous membrane was used for filtering. The chromatographic conditions were slightly modified from the approach of Li et al. [15]. Organic acids were determined using a high-performance liquid chromatograph (HPLC) (Agilent Technologies, Santa Clara, CA, USA) equipped with an Agilent 1260 VWD detector and a reverse phase column (Agilent ZORBAX SB-Aq). The parameters were as follows: KH_2_PO_4_ (0.02 mol/L, pH 2.2) and methanol were used as the mobile phase, the flow rate was set at 0.8 mL/min, the injection volume was 10 μL, the column temperature was kept at 35 °C, and the UV detection wavelength was 210 nm.

### 2.5. Determination of Volatile Flavor Compounds

The extraction of volatile flavor substances from chili bean paste was performed according to the method by Liu et al. [16].and then analyzed by GC-MS using a DB-Wax (30 m × 0.25 mm × 0.25 μm) flexible quartz capillary column (Shanghai Titan Scientific Co., Ltd., Shanghai, China). The initial column temperature of 40 °C was held for 3 min, then increased to 250 °C at 10 °C/min, and held at 250 °C for 6 min. The carrier gas was high purity He (99.999%), the flow rate was 1.0 mL/min, the ion source was an EI source, and the ion source temperature and quadrupole temperature were 200 and 150 °C, respectively. The n-alkanes from C6–C26 were analyzed under the same chromatographic conditions as the samples, and the retention index (RI) of each substance was calculated using the instrument’s operating software. Relative odor activity values (ROAV) were calculated according to the method of Xie et al. [17] 

### 2.6. DNA Extraction, PCR Amplification, and Illumina MiSeq Sequencing

The CTAB method was used to extract the total DNA from the chili bean paste samples, and 1% agarose gel electrophoresis was used to check the DNA purity. The upstream primer (5′-GTGCCAGCMGCCGCGGTAA-3′) and the downstream primer (5′-GGACTACHVGGGTWTCTAAT-3′) were used to amplify the 16S rDNA V4 region. The upstream primer for the fungal ITS region was (5′-GGAAGTAAAAGTCGTAACAAGG-3′) and the downstream primer was (5′-GCTGCGTTCTTCATCGATGC-3′). The PCR reaction mix was diluted to a final volume of 25 μL and contained 2.5 µL of TransStart Buffer, 2.5 µL of dNTP, 2 × 1 µL of primers, 0.5 µL of TransStart Taq DNA, 20 ng of template DNA, and ddH2O. The first-round PCR product was used as a template for the second-round PCR amplification via PCR. The amplification procedure was as follows: denaturation at 98 °C for 1 min; 30 cycles of denaturation at 98 °C for 10 s, annealing at 50 °C for 30 s, and extension at 72 °C for 30 s; a final extension was made at 72 °C for 5 min. The PCR products were identified by 1% agarose gel electrophoresis.

Libraries were constructed using a TruSeq^®^ DNA PCR-free sample-preparation kit (Illumina, San Diego, CA, USA). DNA libraries were validated using an Agilent 2100 bioanalyzer and quantified using a Qubit@ 2.0 fluorometer (Invitrogen, Carlsbad, CA, USA). The library was sequenced on an Illumina NovaSeq platform, and 250-bp paired-end reads were generated.

### 2.7. Data Analysis

SPSS 26.0 software (SPSS Inc. Chicago, IL, USA) was used to evaluate significant differences (*p* < 0.05). The orthogonal projections to the latent structures–discriminant analysis (OPLS-DA) and variable importance of projection (VIP) were performed using SIMCA 14.1 software (Umetrics, Umea, Sweden). OPLS-DA is a supervised pattern-classification and -identification method that can accurately characterize intergroups between samples [18]. The VIP value can reflect the extent to which variables contribute to the model [19].

Spearman’s correlation was used to analyze flavor compounds and microorganisms (|r| > 0.6, *p* < 0.05). Spearman’s correlation is a non-parametric, hierarchical correlation measure that measures the trend correlation between variables, i.e., whether if one variable increases, the other variable will also increase or decrease accordingly. Correlations were visualized using Cytoscape software (version 3.9.1).

## 3. Results

### 3.1. Analysis of Basic Nutrition

**Figure 1:**
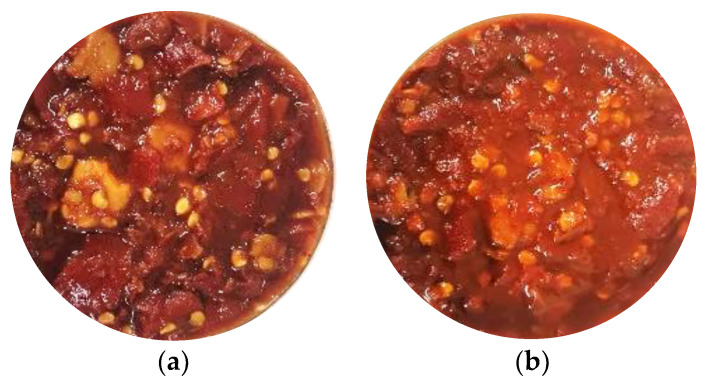
Images of chili bean paste before (**a**) and after (**b**) post-fermentation.

Post-ripening fermentation has a notable impact on the quality of chili bean paste. BP6, in comparison to BP0, exhibited a heightened reddish coloration, a moderate increase in viscosity, a more pronounced sauce-ester aroma, and a mellower taste (Figure 1). With extended fermentation time, the pH decreased and the total acid content increased (Table 1). This was because microorganisms utilized nutrients, such as sugars in raw materials, to metabolize and produce a large amount of organic acids [20]. The decrease in pH is not only conducive to the formation of flavor, but also inhibits the growth of spoilage bacteria, thus extending the storage life of products.

The content of reducing sugar increased significantly from 1.29 g/100 g to 3.14 g/100 g during post-ripening fermentation (*p* < 0.05). The production of reducing sugars was caused by the action of glycoside hydrolase during fermentation, which participates in the Maillard reaction to promote the formation of color and flavor compounds [21]. The protein and amino acid nitrogen contents of the samples increased significantly by 21.74% and 47.83%, respectively. The proteins were degraded into polypeptides and amino acids by microbial proteases, which not only enriches the flavor, but also improves the nutritional value of the product [22,23], such as amino acid nitrogen, an important index for judging the fermentation maturity and flavor quality of bean paste [6,24]. The amino acid nitrogen content of BP6 was 0.34 g/100 g, which reached the premium standard of bean paste [8] (≥0.25 g/100 g) and was close to that of bean paste fermented for 36 months (0.36 g/100 g) [3]. These results indicate that post-ripening fermentation can effectively improve the quality of chili bean paste and the quality of the product to meet the evaluation criteria.

### 3.2. Analysis of Free Amino Acids

Free amino acids can form volatile flavor components, such as aldehydes, ketones, esters, and pyrazines, that are crucial for the distinctive flavor characteristics of fermented products, via the Maillard reaction and the Strecker amino acid-degradation reaction [25]. The content of total free amino acids (17.03 mg/g dw) and essential amino acids (7.01 mg/g dw) in BP6 were significantly higher than those in BP0 (*p* < 0.05) (Table 2). Among the 17 free amino detected, except for Cys, the content of most free amino acids increased during post-ripening fermentation. Glu (4.00 mg/g dw) was the most abundant amino acid and its content increased by 55.04% during fermentation. It has been reported that the glutaminase of *Lactiplantibacillus* can promote the accumulation of Glu and further increase the umami taste of fermented food [26].

**Figure 2:**
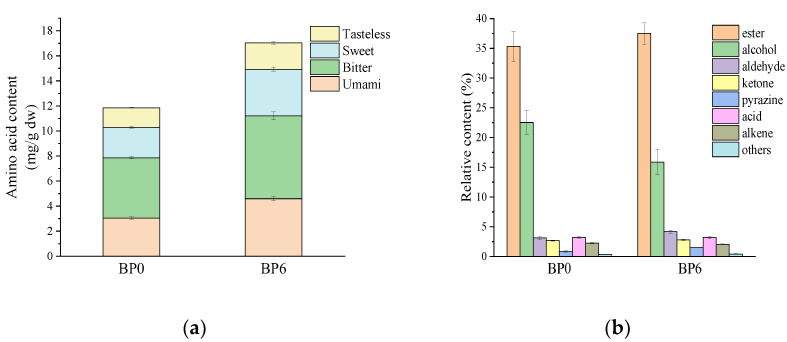
Taste characteristics of free amino acids (**a**) and relative content of volatile substances (**b**) in chili bean paste.

Free amino acids can be classified according to the intensity of taste as umami amino acids, sweet amino acids, bitter amino acids, and tasteless amino acids, among which umami amino acids and bitter amino acids are the main flavor-presenting amino acids in chili bean paste (Figure 2a). These two flavor-presenting amino acids accounted for 65.88% of total free amino acids in BP6. The content of flavor-presenting amino acids increased throughout post-ripening fermentation. The contribution of amino acids to food taste is related to the concentrations and thresholds of amino acids. TAV > 1 was considered a major contributor to food flavor. Amino acids with TAV > 1 in BP6 included Glu (80.00), Asp (19.67), Val (2.58), Lys (2.40), His (1.95), Ala (1.90), Arg (1.70), and Ile (1.07). The TAV of Asp and Glu were much higher than that of the other amino acids and were the main source of umami. Therefore, chili bean paste is usually used as a freshness enhancer in condiments.

### 3.3. Analysis of Organic Acids

Organic acids are important flavor components, and their composition and content are affected by microbial populations and raw materials [27,28]. Six organic acids were detected in BP6 (Table 3). The content of citric acid, malic acid, and tartaric acid were significantly increased (*p* < 0.05), among which citric acid (5.43 g/kg) was the highest (1.51 times). This was consistent with the research results showing that citric acid dominated the post-ripening fermentation process of bean paste [27]. Citric acid is a key product of the tricarboxylic acid cycle [29]. and can be used as an acidity regulator to affect the bean-paste system and promote the formation of special flavors. Lactic acid and malic acids also provide chili bean paste with a more harmonious, softer, refreshing, and pleasant taste. They can optimize and improve product qualities through microorganism biosynthesis, such as -*Lactobacillus*, *Rhizopus oryzae*, etc. [30,31].

### 3.4. Analysis of Volatile Flavor Compounds

A total of 103 volatile compounds were detected in the chili bean paste, including 33 esters, 15 alcohols, 10 aldehydes, 15 ketones, 12 acids, 6 pyrazines, 10 alkenes, and 2 other classes of compounds (Table S1), among which esters were the most abundant flavor compounds during post-ripening fermentation, followed by alcohols and aldehydes (Figure 2b). This observed trend aligned with previously reported findings by Lan et al. [32]. Moreover, distinct differences in the relative contents of various volatile compounds were observed between BP0 and BP6. With an extended fermentation time, the ester content of the chili bean paste increased and the alcohol content decreased. Alcohols are important precursors of esters, and can be esterified and dehydrated with acids under the action of esterifying enzymes to produce esters, bestowing the chili bean paste with a sweet and fruity flavor [28].

**Figure 3:**
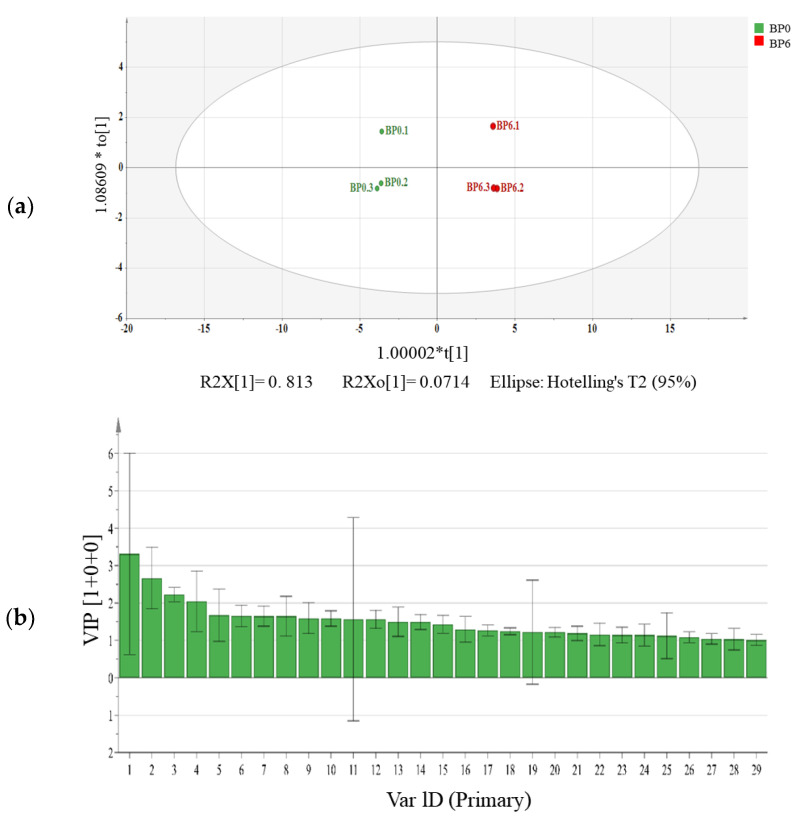
OPLS-DA score plots (**a**) and variable importance in projection (VIP) values (**b**) of volatile flavor substances in chili bean paste. (1) 2−octanol, (2) Methyl salicylate, (3) Benzaldehyde, (4) Methyl tetradecanoate, (5) Benzeneethanol, (6) 2,3,5,6−tetramethyl pyrazine, (7) Methyl palmitoleate, (8) 1−hydroxy−3−methylbutane, (9) Dodecanoic acid, methyl ester, (10) Pentanoic acid, (11) Hexadecanoic acid, methyl ester, (12) 3−hexen−1−ol, (13) 3−methylbutanoic acid, (14) Ethyl tetradecanoate, (15) (2s)−2−methylbutanal, (16) (E,E)−methyl linolelaidate, (17) 1,4−bis(formyloxy) −2−butanone, (18) 2−methyltetradecane, (19) 9−octadecenoic acid, methyl ester, (E)−, (20) Methyl citronellate, (21) 3,5−heptadien−2-one, 6−methyl−, (E)−, (22) Propanoic acid, (23)−A−selinene, (24) 3-pentanone, (25) Hirsutene, (26) Tetramethylpyrazine, (27) Ethyl laurate, (28) (E,E)−2,6−dimethyl−1,3,5,7−octatetraene, and (29) 2−acetyl−1−methylcyclopentene.

To further analyze the differences in volatile flavor substances between BP0 and BP6, we established an OPLS-DA model, used variable projection (VIP) to evaluate the contribution of variables, and screened for flavor substances with a VIP > 1 and *p* < 0.05 (Figure 3). BP0 and BP6 were well separated, indicating a large difference before and after fermentation (Figure 3a). A total of 29 major volatile differential substances were screened, including 2−octanol, methyl salicylate, benzeneethanol, 2,3,5,6−tetramethyl pyrazine, etc. (Figure 3b). 2−Octanol and benzeneethanol had relatively high VIP values and were widely present in the fermented bean paste, which can provide sweet and flower aromas to the products [2]. Studies have shown that benzeneethanol is produced mainly through the phenylalanine metabolism pathways and the biosynthesis of plant secondary metabolites [33]. Methyl salicylate is a phenol derivative with a mint flavor and has been considered a key flavor metabolite in chili sauce [34]. 2,3,5,6−Tetramethyl pyrazine was a new volatile flavor compound in BP6, which can be produced by the Maillard reaction, and bestows a nutty flavor to chili bean paste [35]. These results showed that the post-ripening fermentation of chili bean paste contributed to the formation of characteristic flavors.

The relative odor-activity value (ROAV) was used to evaluate the contribution of volatile flavor compounds to the product aroma. ROAV > 1 indicated that the substance could be judged as a characteristic flavor substance and contributed to the overall flavor. Fifteen characteristic flavor substances were screened during the post-ripening fermentation of chili bean paste using ROAV > 1 as a criterion (Table 4), among which (E)−2−octenal, (E)−2−heptenal, 2−octanol, and linalool oxide had high ROAV values. These compounds exhibited fruity, citrus, and floral aromas that harmonized the overall flavor of the chili bean paste. Notably, 4-vinylphenol was detected in chili bean paste, which is the main source of odor for spicy flavors and is commonly found in fermented red chilies [5,36]. The flavors of the chili bean paste were diverse and complex during the post-ripening fermentation. It has been found that flavor substances in fermented foods are closely related to microbial metabolism. Therefore, to further elucidate the relationship between flavor substances and microbial metabolism in chili bean paste, the microbial community succession was investigated.

### 3.5. Microbial-Community Composition

A total of 386,360 16S rDNA and 443,161 ITS rDNA valid sequences were obtained from the chili bean paste, of which 1912 bacterial and 1743 fungal OTUs were identified at a concordance threshold of 97%. As shown in Table S2, Shannon and Simpson indices showed that the bacterial diversity in chili bean paste increased while fungal diversity decreased. Both Simpson and Shannon indices of bacteria in BP6 were higher than those of fungi. In addition, the Chao1 and ACE indices of bacteria and fungi were reduced, which may be related to the competition of dominant strains during fermentation.

**Figure 4:**
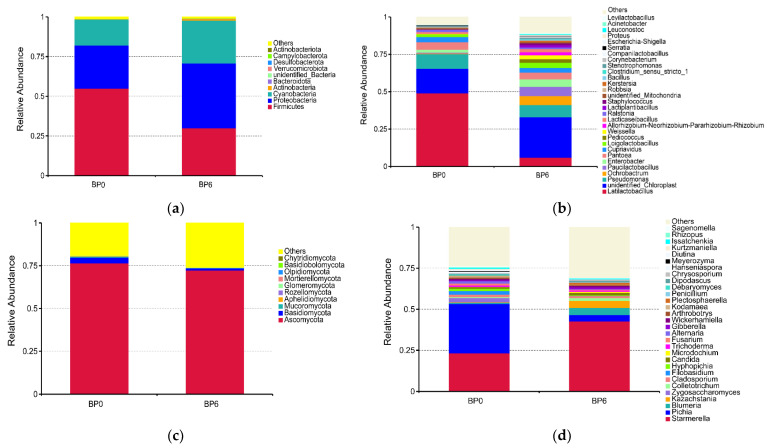
Relative abundance of bacteria from chili bean paste at the phylum level (**a**) and genus level (**b**). Relative abundance of fungi from chili bean paste at the phylum level (**c**) and genus level (**d**).

A total of 18 bacterial phyla were identified in the samples. The main phyla were Firmicutes (54.98%), Proteobacteria (27.10%), and Cyanobacteria (16.43%) (Figure 4a). Furthermore, 232 bacterial genera were detected, mainly *Latilactobacillus*, *Paucilactobacillus*, *Enterobacter*, *Pantoea*, *Weissella*, *Loigolactobacillus*, and *Lacticaseibacillus*, etc. (Figure 4b). These results were similar to those of previous reports [27,37]. The relative abundances of *Weissella* and *Lacticaseibacillus* were increased. These genera participate in glucose metabolism and produce organic acids that give the product its special flavor [38]. However, the relative abundance of *Latilactobacillus* decreased from 48.96% to 5.94% during post-ripening fermentation, likely due to other microorganisms competing with *Latilactobacillus* for nutrients. It is worth noting that *Pseudomonas* was detected in the chili bean paste. This is a strictly aerobic bacterium that may produce a variety of alcohols and break down fats and proteins to produce precursors for certain volatile flavor compounds, and is widely distributed in fermented foods such as chopped red chili, paocai [39,40,41]. However, Pseudomonas is commonly considered a spoilage or parasitic pathogen [42]. Therefore, in an open-processing environment, we need to pay attention to the sources of spoilage bacteria in fermented foods and take effective preventive and control measures to reduce the safety risks. Microbial competition mechanisms, artificial inoculation, or other methods can be used to regulate the growth of strains to prevent the growth of spoilage bacteria or pathogenic bacteria, ensure product safety, and further improve the flavor and texture of the product.

Of the fungal communities, Ascomycora (76.47%) and Basidiomycota (3.33%) were the main phylum in chili bean paste (Figure 4c). A total of 198 fungal genera were detected, of which *Pichia*, *Starmerella*, *Zygosaccharomyces*, *Cladosporium*, *Hyphopichia*, *Kazachstania*, and *Candida* were the dominant genera (Figure 4d). The relative abundance of Pichia decreased significantly from 30.12% to 3.89%, which was similar to the results of Yang et al. [43]; thus, it is speculated that the large-scale death of *Pichia* occurs during the long ripening process. The relative abundance of *Kazachstania* and *Candida* increased, which can produce octanoic acid ethyl ester, 1-octanol, and other aromatically active compounds [44]. In addition, it has been revealed that a low relative abundance of yeasts and molds such as *Issatchenkia*, *Hyphopichia*, *Penicillium*, and *Rhizopus* can also affect the flavor of the product [39,45].

### 3.6. Microbial Interactions at the Genus Level

**Figure 5:**
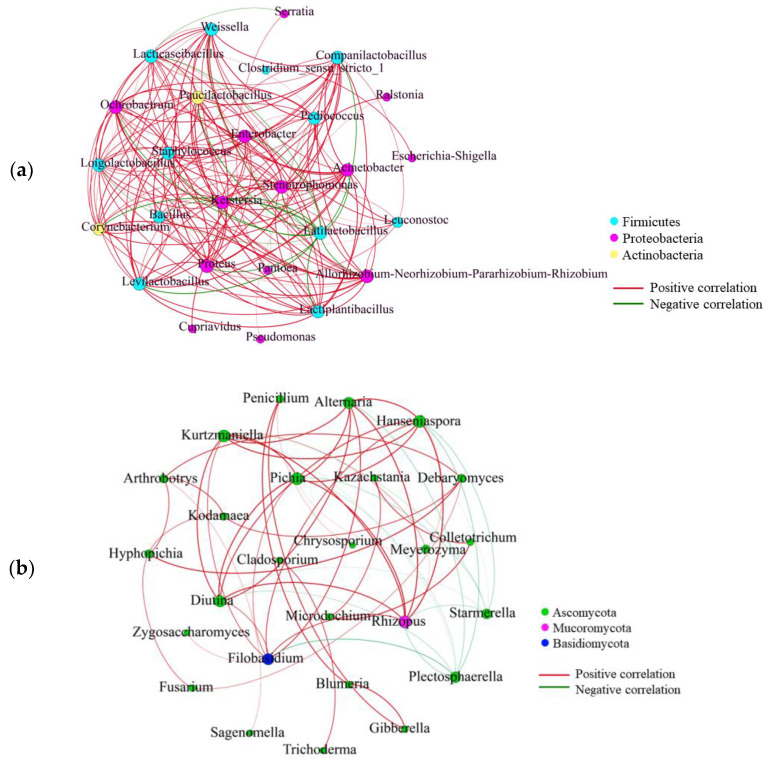

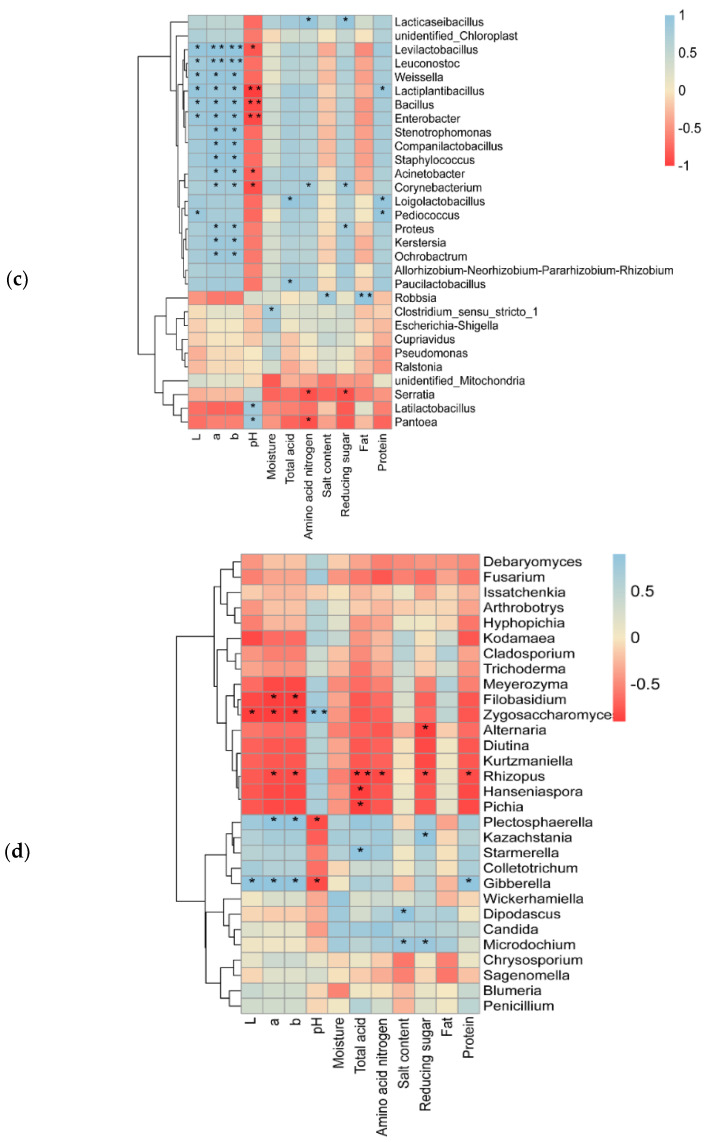
Pearson correlation between bacterial groups (**a**) and fungal groups (**b**) at the genus level. A connection stands for a significant correlation (|r| > 0.6, *p* < 0.05). The node size is related to the weighting degree. The larger the weighting degree, the larger the node. The red lines represent positive correlations while the green lines represent the negative correlation. Heatmap of Spearman correlation between the genera and physicochemical properties in chili bean paste before and after post−fermentation, where (**c**) stands for bacteria genera and (**d**) stands for fungi genera. * represents the correlation between the genera and physicochemical properties. (* stands for *p* < 0.05; ** stands for *p* < 0.01).

To explore the microbial interaction during the post-ripe fermentation of chili bean paste, a Pearson correlation (|r| > 0.6) was used to analyze the positive and negative correlations among the top 30 dominant bacterial genera by abundance (Figure 5a,b). Positive and negative correlations represented potential symbiotic and antagonistic relationships between genera. The results of the correlation analysis showed that *Lactiplantibacillus*, *Paucilactobacillus*, *Enterobacter*, *Pediococcus*, and *Weissella* were positively correlated with each other (Figure 5a). These genera of bacteria are key contributors in the production of fermented foods such as soy sauce and cheese [46]. However, *Latilactobacillus* was negatively correlated with *Paucilactobacillus*, *Enterobacter*, *Loigolactobacillus*, *Weissella*, *Lacticaseibacillus*, and *Lactiplantibacillus*, suggesting that the growth and metabolism of *Latilactobacillus* and other bacterial genera may have been mutually inhibited. This could explain the aforementioned decrease in the relative abundance of *Latilactobacillus*. In addition, *Lacticaseibacillus* was negatively correlated with *Pantoea* and *Serratia*. *Lacticaseibacillus* inhibits the growth of other microorganisms by producing organic acids and antibiotics, and competes for nutrients [47]. Li et al. [48] also demonstrated the important role of community composition and the quantity of microorganisms in low-salt fermented-red-pepper sauce. *Levilactobacillus*, *Companilactobacillus* and *Candida* were the dominant genera, and *Levilactobacillus* was significantly negatively correlated with *Lactiplantibacillus*, *Weissella* and *Enterococcus*. Except for *Plectosphaerella* and *Starmerella*, which were negatively correlated with other fungal genera, most fungal genera were positively correlated (Figure 5b). *Rhizopus* was positively correlated with *Pichia*, *Filobasidium*, *Alternaria*, and *Hanseniaspora*. Interactions between bacterial communities were stronger than those in the fungal communities in the chili bean paste. The interaction between these microorganisms constitutes the biological regulatory mechanism in the fermentation process, which maintains the natural steady-state fermentation and dynamic equilibrium of the entire fermentation system.

### 3.7. Correlation Analysis of Microbial and Physicochemical Indicators

The correlations between the dominant bacterial and fungal genera (top 30 genera) and physicochemical indices during the post-ripening fermentation of chili bean paste are shown in Figure 5c,d. At the bacterial genus level (Figure 5c), *Lactiplantibacillus*, *Bacillus*, *Enterobacter*, *Acinetobacter*, *Corynebacterium*, and *Levilactobacillus* showed a significant negative correlation with pH. *Lacticaseibacillus* was significantly positively correlated with amino acid nitrogen and reducing sugars. *Lacticaseibacillus* converts starch to glucose via an amylase enzyme [49]. Most of the dominant bacterial genera were positively correlated with color, indicating that microbial action had an important effect on the color of chili bean paste. The correlation between fungi and the physicochemical indicators of the chili bean paste was in sharp contrast to that of bacteria (Figure 5d). *Rhizopus*, *Hanseniaspora*, and *Pichia* were significantly negatively correlated with total acid, while positively correlated with pH (*p* < 0.05). *Zygosaccharomyces* is the main genus of fungi tolerant to low pH during the development of bean paste [3]. In the present study, *Zygosaccharomyces* was found to have a significant positive correlation with pH (*p* < 0.05), which could be explained by the alcoholic fermentation. *Candida* ferments glucose and is positively correlated with reducing sugars, and was the dominant fungus affecting flavor production [50].

### 3.8. Correlation Analysis of Microbial and Flavor Compounds

**Figure 6:**
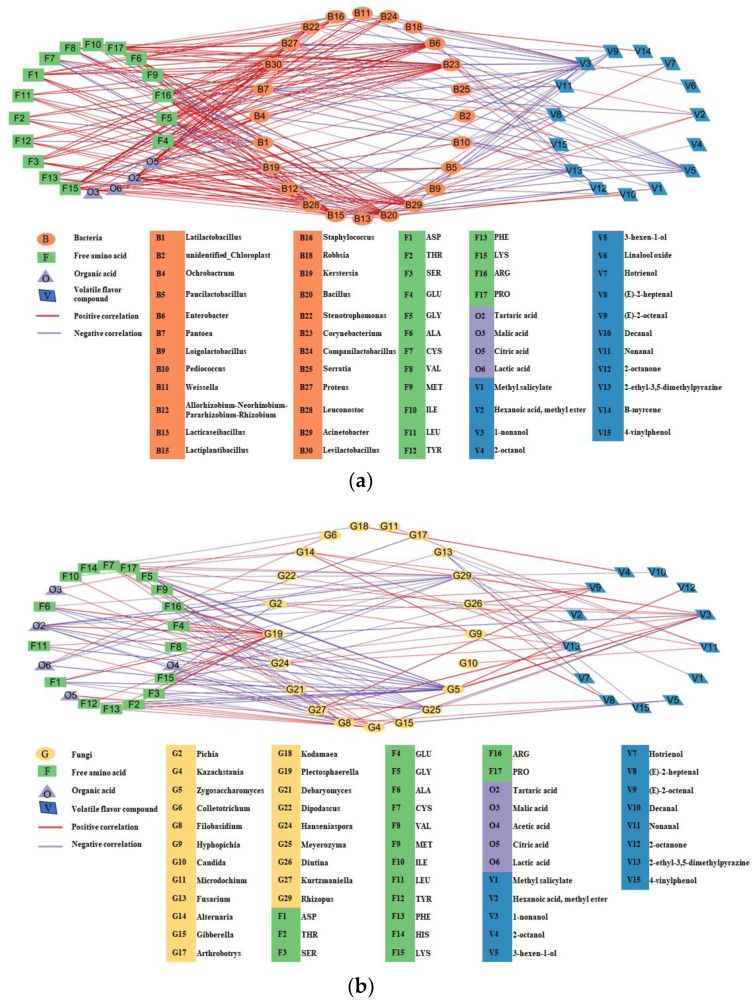
The network correlation between microbial genera and flavor compounds in chili bean paste. (**a**) stands for bacteria genera; (**b**) stands for fungi genera. Red and violet lines represent positive and negative correlation coefficients, respectively. (|r| > 0.6, *p* < 0.05).

During the post-ripening fermentation of chili bean paste, we found that 20 bacterial genera and 15 fungal genera were associated with organic acids, 20 bacterial genera and 12 fungal genera were associated with free amino acids, and 23 bacterial genera and 18 fungal genera were associated with characteristic flavor substances (ROAV > 1) (|r| > 0.6, *p* < 0.05) (Figure 6). Among the bacterial genera (Figure 6a), *Lacticaseibacillus* and *Weissella* were significantly and positively correlated with organic acids such as malic acid and citric acid (r > 0.8, *p* < 0.05), mainly through participation in the tricarboxylic acid cycle [51]. This pathway promotes the synthesis of malic acid and citric acid, and makes the taste of chili bean paste more mellow. Free amino acids can improve taste through proteolysis and are closely related to proteases secreted by various microorganisms [7]. The results of this study showed that *Enterobacter*, *Lactiplantibacillus*, *Bacillus*, *Corynebacterium*, *Leuconostoc*, and *Acinetobacter* were highly correlated with more than 10 free amino acids (|r| > 0.8, *p* < 0.05). Glu, which contributed the most to the flavor of broad bean paste, was significantly positively correlated with *Enterobacter*, *Bacillus*, *Lactiplantibacillus*, and *Leuconostoc* (r > 0.9, *p* < 0.05). It has been shown that some species of *Bacillus* can produce serine alkaline proteases, metalloproteases, and serine proteases, which play an important role in the formation of bean paste flavor through amino acid metabolism [52,53]. In addition to amino acid metabolism, carbohydrate metabolism is also an important pathway for producing volatile flavor substances [54]. Lactic acid bacteria produces lactic acid through the breakdown of carbohydrates, which is an important substrate for the formation of esters [55]. For example, *Lacticaseibacillus* was significantly positively correlated with methyl salicylate, hexanoic acid, methyl ester, hotrienol, nonanal, and 4-vinylphenol (r > 0.8, *p* < 0.05). In addition, hexanoic acid, methyl ester, decanal, and 4-vinylphenol were significantly positively associated with *Lactiplantibacillus* (r > 0.8, *p* < 0.05). The results showed that lactic acid bacteria had a positive effect on the flavor quality of chili bean paste.

Some fungi were closely related to the flavor substances of the chili bean paste such as *Pichia*, *Kazachstania*, *Alternaria, Hanseniaspora*, *Diutina*, *Kurtzmaniella*, *Rhizopus*, etc. (Figure 6b). *Kazachstania* was found to be significantly and positively correlated with citric acid and several free amino acids (r > 0.6, *p* < 0.05). *Pichia*, *Alternaria*, *Rhizopus*, and *Kurtzmaniella* were significantly positively correlated with 1-nonanol and 2-ethyl-3,5-dimethylpyrazine (r > 0.8, *p* < 0.05). Some studies have shown that *Pichia* can use glucose to produce a large number of volatile flavor substances, such as phenylethanol, 3-methylbutanoic acid, and ethyl linoleate, etc. [56]. *Rhizopus* promotes the production of amylase, which can break down starch into glucose and is metabolized to produce alcohols, acids, and other flavor substances [45]. In addition, 1-nonanol and 2-octanone were significantly positively correlated with *Zygosaccharomyces* (r > 0.8, *p* < 0.05). *Candida* was significantly positively correlated with Nonanal (r > 0.8, *p* < 0.05). Compared with fungi, more bacterial genera had a higher correlation with flavor compounds (r > 0.6, *p* < 0.05), indicating that bacteria may be the dominant microorganisms in the formation of chili bean paste’s flavor.

## 4. Conclusions

This study revealed significant differences in flavor compounds (6 organic acids, 17 free amino acids and 103 volatile flavor compounds) and microbial communities (263 bacterial and 380 fungal genera) before and after post-ripening fermentation. Tartaric acid, malic acid, and citric acid increased significantly during the post-ripening fermentation of chili bean paste (*p* < 0.05). Free amino acids content increased after fermentation, especially the content of Glu. Esters, aldehydes, and alcohols contributed the most to the flavor of the chili bean paste. *Latilactobacillus*, *Starmerella*, and *Pichia* were found to be the dominant microbial genera in the fermented samples. Microbial communities, including *Corynebacterium*, *Bacillus*, *Enterobacter*, and *Lactiplantibacillus*, were the most important microbial genera contributing to amino acid formation. This study provided theoretical insights into the microbial communities and flavor components of traditional Chinese chili bean paste, thereby potentially informing its production optimization.

## Figures and Tables

**Table 1:** Analysis of physicochemical characterization in chili bean paste.

	BP0	BP6
L	10.94 ± 0.54 ^b^	15.58 ± 0.62 ^a^
a	1.68 ± 0.08 ^b^	9.73 ± 0.72 ^a^
b	1.92 ± 0.19 ^b^	3.00 ± 0.12 ^a^
pH	4.98 ± 0.01 ^a^	4.89 ± 0.06 ^a^
Moisture (%)	65.57 ± 1.84 ^a^	66.64 ± 2.11 ^a^
Total acid (g/100 g)	0.55 ± 0.05 ^b^	0.80 ± 0.08 ^a^
Amino acid nitrogen (g/100 g)	0.23 ± 0.02 ^b^	0.34 ± 0.03 ^a^
Salt content (g/100 g)	13.18 ± 0.39 ^a^	13.42 ± 0.73 ^a^
Reducing sugar (g/100 g)	1.29± 0.13 ^b^	3.14± 0.07 ^a^
Fat (g/100 g dw)	0.44 ± 0.03 ^a^	0.44 ± 0.05 ^a^
Protein (g/100 g dw)	7.45 ± 0.13 ^b^	9.07 ± 1.18 ^a^

Note: different lowercase letters in the same row represent significant differences between samples (*p* < 0.05).

**Table 2:** Types and contents of free amino acids in chili bean paste.

Taste	FAA	BP0 (mg/g)	BP6 (mg/g)	Threshold (mg/g)	TAV	
					BP0	BP6
Umami	ASP	0.47 ± 0.01 ^b^	0.59 ± 0.02 ^a^	0.03	15.67	19.67
	GLU	2.58 ± 0.08 ^b^	4.00 ± 0.15 ^a^	0.05	51.60	80.00
Sweet	THR	0.46 ± 0.01 ^b^	0.75 ± 0.03 ^a^	2.6	0.18	0.29
	SER	0.82 ± 0.02 ^b^	1.26 ± 0.05 ^a^	1.5	0.55	0.84
	GLY	0.35 ± 0.01 ^b^	0.56 ± 0.02 ^a^	1.3	0.27	0.43
	ALA	0.80 ± 0.03 ^b^	1.14 ± 0.05 ^a^	0.6	1.33	1.90
Bitter	VAL	0.71 ± 0.02 ^b^	1.03 ± 0.03 ^a^	0.4	1.78	2.58
	PHE	0.67 ± 0.02 ^b^	0.86 ± 0.03 ^a^	0.9	0.74	0.96
	MET	0.12 ± 0.00 ^b^	0.21 ± 0.04 ^a^	0.9	0.13	0.23
	LEU	1.19 ± 0.04 ^b^	1.61 ± 0.08 ^a^	1.9	0.63	0.85
	ILE	0.77 ± 0.04 ^a^	0.96 ± 0.13 ^a^	0.9	0.86	1.07
	HIS	0.33 ± 0.06 ^a^	0.39 ± 0.01 ^a^	0.2	1.65	1.95
	ARG	0.55 ± 0.02 ^b^	0.85 ± 0.03 ^a^	0.5	1.10	1.70
	TYR	0.46 ± 0.02 ^b^	0.72 ± 0.09 ^a^	0.91	0.51	0.79
Tasteless	CYS	0.02 ± 0.00 ^a^	0.01 ± 0.00 ^b^	-	-	-
	LYS	1.00 ± 0.02 ^b^	1.20 ± 0.08 ^a^	0.5	2.00	2.40
	PRO	0.55 ± 0.01 ^b^	0.89 ± 0.04 ^a^	3	0.18	0.30
TAA		11.85 ± 0.24 ^b^	17.03 ± 0.55 ^a^			
EAA		5.26 ± 0.07 ^b^	7.01 ± 0.21 ^a^			
EAA/TAA		44.35 ± 0.43 ^a^	41.19 ± 0.96 ^b^			

Note: indicators in the table are presented as mean ± standard deviation (mg/g dw). Different lowercase letters in the same row indicate significant differences between groups (*p* < 0.05). TAA stands for total free amino acids; EAA stands for essential amino acids; EAA/TAA (%); - means not detected.

**Table 3:** Analysis of organic acids in chili bean paste.

Sample	Oxalic Acid	Tartaric Acid	Malic Acid	Acetic Acid	Citric Acid	Lactic Acid
BP0	0.48 ± 0.09 ^a^	0.43 ± 0.03 ^b^	0.25 ± 0.05 ^b^	0.55 ± 0.07 ^a^	3.60 ± 0.94 ^b^	0.88 ± 0.09 ^a^
BP6	0.47 ± 0.08 ^a^	0.84 ± 0.06 ^a^	0.69 ± 0.14 ^a^	0.62 ± 0.07 ^a^	5.43 ± 0.25 ^a^	1.00 ± 0.11 ^a^

Note: different lowercase letters in the same column represent significant differences between samples (g/kg) (*p* < 0.05).

**Table 4:** Analysis of relative odor-activity value (ROAV) of volatile compounds in chili bean paste.

Flavor Compounds	Threshold	Odor Description	ROAV		Relative Content (%)	
			BP0	BP6	BP0	BP6
Methyl salicylate	0.06	Oil and grease aroma	2.04	11.00	0.49 ± 0.10	2.64 ± 0.50
Hexanoic acid, methyl ester	0.01	-	3.50	7.00	0.14 ± 0.03	0.28 ± 0.03
1-nonanol	0.02	Fatty aroma, floral	1.88	-	0.15 ± 0.02	-
2-octanol	0.12	Oily, citrusy	31.48	23.69	15.11 ± 1.77	11.37 ± 2.05
3-hexen-1-ol	0.07	Wine, fruit flavors	3.61	1.00	1.01 ± 0.04	0.28 ± 0.05
Linalool oxide	0.006	Woody, floral aroma	13.75	12.50	0.33 ± 0.06	0.30 ± 0.01
Hotrienol	0.006	Floral, citrusy	2.08	4.17	0.05 ± 0.01	0.10 ± 0.01
(E)-2-heptenal	0.0005	Grassy aroma (fruity)	40.00	25.00	0.08 ± 0.00	0.05 ± 0.00
(E)-2-octenal	0.0002	Cucumber, vegetable flavor	100.00	87.50	0.08 ± 0.00	0.07 ± 0.01
Decanal	0.005	Aromatic, fruity, floral	2.00	3.00	0.04 ± 0.00	0.06 ± 0.01
Nonanal	0.015	Oily and fruity aroma	2.83	3.50	0.17 ± 0.03	0.21 ± 0.03
2-octanone	0.041	Floral fragrance	3.54	1.83	0.58 ± 0.10	0.30 ± 0.05
2-ethyl-3,5-dimethylpyrazine,	0.00025	Caramel	50.00	-	0.05 ± 0.00	-
Β-myrcene	0.042	Floral fragrance	1.01	0.89	0.17 ± 0.03	0.15 ± 0.03
4-vinylphenol	0.02	Caramel, spicy aroma	1.63	5.13	0.13 ± 0.01	0.41 ± 0.07

Note: thresholds from L. J. van Gemert’s «Compilation of compound aroma thresholds».

## Data Availability

The original contributions presented in the study are included in the article/Supplementary Materials, further inquiries can be directed to the corresponding author.

## Supplementary Materials

The following supporting information can be downloaded at https://www.mdpi.com/article/10.3390/foods13081209/s1. Figure S1: HPLC (a) and GC-MS (b) chromatograms of BP6; Table S1: The relative content of flavor compounds in post-fermentation period of chili bean paste; Table S2: Bacterial and fungal alpha diversity of chili bean paste.

## References

[1] Liu C., Zhu T., Song H., Niu C., Wang J., Zheng F., Li Q. (2021). Evaluation and prediction of the biogenic amines in Chinese traditional broad bean paste. J. Food Sci. Tech. Mys..

[2] Yang M., Huang J., Zhou R., Qi Q., Peng C., Zhang L., Jin Y., Wu C., Tang Q. (2020). Characterization of the flavor in traditional Pixian Doubanjiang by polyphasic quantitative detection technology. Food. Res. Int..

[3] Zhang L., Che Z., Xu W., Yue P., Li R., Li Y., Pei X., Zeng P. (2020). Dynamics of physicochemical factors and microbial communities during ripening fermentation of Pixian Doubanjiang, a typical condiment in Chinese cuisine. Food Microbiol..

[4] Lu Y., Tan X., Lv Y., Yang G., Chi Y., He Q. (2020). Physicochemical properties and microbial community dynamics during Chinese horse bean-chili-paste fermentation, revealed by culture-dependent and culture-independent approaches. Food Microbiol..

[5] Li Z., Dong L., Huang Q., Wang X. (2016). Bacterial communities and volatile compounds in Doubanjiang, a Chinese traditional red pepper paste. J. Appl. Microbiol..

[6] Lin H., Bi X., Zhou B., Fang J., Liu P., Ding W., Che Z., Wang Q., He Q. (2021). Microbial communities succession and flavor substances changes during Pixian broad-bean paste fermentation. Food Biosci..

[7] Yang M., Huang J., Zhou R., Qi Q., Peng C., Zhang L., Jin Y., Wu C. (2021). Characterizing the microbial community of Pixian Doubanjiang and analysing the metabolic pathway of major flavour metabolites. Lwt-Food Sci. Technol..

[8] (2006). Geographical Indication Products, Pixian Douban.

[9] (2006). National Standard for Food Safety Food, Determination of Moisture in Food.

[10] Kim M.K., Chung H., Bang W. (2018). Correlating physiochemical quality characteristics to consumer hedonic perception of traditional Doenjang (fermented soybean paste) in Korea. J. Sens. Stud..

[11] (2016). National Standard for Food Safety, Determination of Fat in Food.

[12] Zhang L., Zhou R., Cui R., Huang J., Wu C. (2016). Characterizing Soy Sauce Moromi Manufactured by High-Salt Dilute-State and Low-Salt Solid-State Fermentation Using Multiphase Analyzing Methods. J. Food Sci..

[13] Xu Z., Zhong D., Wang J., Mei Q., Wu Q., Qin L., Zeng H. (2023). Colour, physicochemical, microbiological and bioactive component quality analysis of peppers in Guizhou. Int. J. Food Sci. Tech..

[14] Tian Z., Zhu Q., Chen Y., Zhou Y., Hu K., Li H., Lu K., Zhou J., Liu Y., Chen X. (2022). Studies on Flavor Compounds and Free Amino Acid Dynamic Characteristics of Fermented Pork Loin Ham with a Complex Starter. Foods.

[15] Li J., Wang X., Wu W., Jiang J., Feng D., Shi Y., Hu P. (2022). Comparison of Fermentation Behaviors and Characteristics of Tomato Sour Soup between Natural Fermentation and Dominant Bacteria-Enhanced Fermentation. Microorganisms.

[16] Liu N., Pan J., Miao S., Qin L. (2020). Microbial community in Chinese traditional fermented acid rice soup ( rice-acid ) and its correlations with key organic acids and volatile compounds. Food Res. Int..

[17] Xie C., Zeng H., Wang C., Xu Z., Qin L. (2018). Volatile flavour components, microbiota and their correlations in different sufu, a Chinese fermented soybean food. J. Appl. Microbiol..

[18] Kang C., Zhang Y., Zhang M., Qi J., Zhao W., Gu J., Guo W., Li Y. (2022). Screening of specific quantitative peptides of beef by LC-MS/MS coupled with OPLS-DA. Food Chem..

[19] Arendse E., Fawole O.A., Magwaza L.S., Nieuwoudt H., Opara U.L. (2018). Evaluation of biochemical markers associated with the development of husk scald and the use of diffuse reflectance NIR spectroscopy to predict husk scald in pomegranate fruit. Sci. Hortic..

[20] Liao H., Luo Y., Huang X., Xia X. (2023). Dynamics of quality attributes, flavor compounds, and microbial communities during multi-driven-levels chili fermentation: Interactions between the metabolome and microbiome. Food Chem..

[21] Ding W., Ye X., Zhao X., Liu Y., Zhang M., Luo Y., Xiong Y., Liu Y., Che Z., Lin H. (2022). Fermentation characteristics of Pixian broad bean paste in closed system of gradient steady-state temperature field. Food Chem..

[22] Ketnawa S., Ogawa Y. (2019). Evaluation of protein digestibility of fermented soybeans and changes in biochemical characteristics of digested fractions. J. Funct. Foods.

[23] Seong H., Kim M. (2021). Enhanced protein quality and antioxidant activity of fermented Brown rice with Gryllus bimaculatus. Lwt-Food Sci. Technol..

[24] Li Z., Rui J., Li X., Li J., Dong L., Huang Q., Huang C., Wang Z., Li L., Xuan P. (2017). Bacterial community succession and metabolite changes during doubanjiang-meju fermentation, a Chinese traditional fermented broad bean (*Vicia faba* L.) paste. Food Chem..

[25] Chen G., Chen Y., Hou Y., Huo Y., Gao A., Li S., Chen Y. (2020). Preparation, characterization and the in vitro bile salts binding capacity of celery seed protein hydrolysates via the fermentation using B. subtilis. Lwt-Food Sci. Technol..

[26] Ruan W., Liu J., Li P., Zhao W., Zhang A., Liu S., Wang Z., Liu J. (2022). Dynamics of Microbial Communities, Flavor, and Physicochemical Properties during Ziziphus jujube Vinegar Fermentation: Correlation between Microorganisms and Metabolites. Foods.

[27] Liu P., Xiang Q., Sun W., Wang X., Lin J., Che Z., Ma P. (2020). Correlation between microbial communities and key flavors during postfermentation of Pixian broad bean paste. Food Res. Int..

[28] Que Z., Jin Y., Huang J., Zhou R., Wu C. (2023). Flavor compounds of traditional fermented bean condiments: Classes, synthesis, and factors involved in flavor formation. Trends Food Sci. Technol..

[29] Gao P., Xia W., Li X., Liu S. (2019). Use of Wine and Dairy Yeasts as Single Starter Cultures for Flavor Compound Modification in Fish Sauce Fermentation. Front. Microbiol..

[30] Wu N., Zhang J., Chen Y., Xu Q., Song P., Li Y., Li K., Liu H. (2022). Recent advances in microbial production of L-malic acid. Appl. Microbiol. Biot..

[31] Zhao Y., Suyama T., Wu Z., Zhang W. (2022). Characterization of variations and correlations between flavor metabolites and microbial communities of industrial paocai brine during fermentation. J. Food Process Pres..

[32] Lan L., Wang J., Wang S., He Q., Wei R., Sun Z., Duan S., Li Y. (2023). Correlation between microbial community succession and flavor substances during fermentation of Yongchuan Douchi. Food Biosci..

[33] Yan Y., Sun L., Xing X., Wu H., Lu X., Zhang W., Xu J., Ren Q. (2022). Microbial succession and exploration of higher alcohols-producing core bacteria in northern Huangjiu fermentation. Amb. Express.

[34] Xu X., Wu B., Zhao W., Lao F., Chen F., Liao X., Wu J. (2021). Shifts in autochthonous microbial diversity and volatile metabolites during the fermentation of chili pepper (*Capsicum frutescens* L.). Food Chem..

[35] Zhao S., Niu C., Xing X., Fan L., Zheng F., Liu C., Wang J., Li Q. (2022). Revealing the changes of microbiota structure and function in broad bean paste mediated by sunlight and ventilation. Lwt-Food Sci. Technol..

[36] Kang K., Baek H. (2014). Aroma quality assessment of Korean fermented red pepper paste (gochujang) by aroma extract dilution analysis and headspace solid-phase microextraction-gas chromatography-olfactometry. Food Chem..

[37] Nam Y., Lee S., Lim S. (2012). Microbial community analysis of Korean soybean pastes by next-generation sequencing. Int. J. Food Microbiol..

[38] Gaenzle M.G. (2015). Lactic metabolism revisited: Metabolism of lactic acid bacteria in food fermentations and food spoilage. Curr. Opin. Food Sci..

[39] Chen Z., Geng Y., Wang M., Du L., Huang S., Guan Y., Hu Y. (2022). Relationship between microbial community and flavor profile during the fermentation of chopped red chili (*Capsicum annuum* L.). Food Biosci..

[40] Wang C., Zhang Q., He L., Li C. (2020). Determination of the microbial communities of Guizhou Suantang, a traditional Chinese fermented sour soup, and correlation between the identified microorganisms and volatile compounds. Food Res. Int..

[41] Wang D., Chen G., Tang Y., Ming J., Huang R., Li J., Ye M., Fan Z., Yin L., Zhang Q. (2022). Effect of non-core microbes on the key odorants of paocai. Lwt-Food Sci. Technol..

[42] Heir E., Moen B., Asli A.W., Sunde M., Langsrud S. (2021). Antibiotic Resistance and Phylogeny of Pseudomonas spp. Isolated over Three Decades from Chicken Meat in the Norwegian Food Chain. Microorganisms.

[43] Yang M., Huang J., Zhou R., Jin Y., Wu C. (2022). Exploring major variable factors influencing flavor and microbial characteristics of Pixian Doubanjiang. Food Res. Int..

[44] Meng Y., Chen X., Sun Z., Li Y., Chen D., Fang S., Chen J. (2021). Exploring core microbiota responsible for the production of volatile flavor compounds during the traditional fermentation of Koumiss. Lwt-Food Sci. Technol..

[45] Chen T., Wang H., Su W., Mu Y., Tian Y. (2023). Analysis of the formation mechanism of volatile and non - volatile flavor substances in corn wine fermentation based on high-throughput sequencing and metabolomics. Food Res. Int..

[46] Feng Y., Wu W., Chen T., Huang M., Zhao M. (2023). Exploring the core functional microbiota related with flavor compounds in fermented soy sauce from different sources. Food Res. Int..

[47] Le Lay C., Coton E., Le Blay G., Chobert J., Haertle T., Choiset Y., Van Long N.N., Meslet-Cladiere L., Mounier J. (2016). Identification and quantification of antifungal compounds produced by lactic acid bacteria and propionibacteria. Int. J. Food Microbiol..

[48] Li Q., Wang Y., Tian Y., Lv L., Dong L., Zhao C., Zhang F., Zuo Y., Zhang S., Li Z. (2023). Dynamic analysis of microbial community, flavor components of low-salt fermented red pepper sauce and exploring the key flavours formation. Food Biosci..

[49] Zhao C., Su W., Mu Y., Jiang L., Mu Y. (2020). Correlations between microbiota with physicochemical properties and volatile flavor components in black glutinous rice wine fermentation. Food Res. Int..

[50] Sakandar H.A., Hussain R., Khan Q.F., Zhang H. (2020). Functional microbiota in Chinese traditional Baijiu and Mijiu Qu (starters): A review. Food Res. Int..

[51] Liu A., Yang X., Guo Q., Li B., Zheng Y., Shi Y., Zhu L. (2022). Microbial Communities and Flavor Compounds during the Fermentation of Traditional Hong Qu Glutinous Rice Wine. Foods.

[52] Ortiz G.E., Noseda D.G., Ponce Mora M.C., Recupero M.N., Blasco M., Albertó E. (2016). A Comparative Study of New Aspergillus Strains for Proteolytic Enzymes Production by Solid State Fermentation. Enzyme Res..

[53] Jeong D., Heo S., Lee B., Lee H., Jeong K., Her J., Lee K., Lee J. (2017). Effects of the predominant bacteria from meju and doenjang on the production of volatile compounds during soybean fermentation. Int. J. Food Microbiol..

[54] Wang P., Mao J., Meng X., Li X., Liu Y., Feng H. (2014). Changes in flavour characteristics and bacterial diversity during traditional fermentation of Chinese rice wines from Shaoxing region. Food Control.

[55] Montanari C., Bargossi E., Gardini A., Lanciotti R., Magnani R., Gardini F., Tabanelli G. (2016). Correlation between volatile profiles of Italian fermented sausages and their size and starter culture. Food Chem..

[56] Ma D., Li Y., Chen C., Fan S., Zhou Y., Deng F., Zhao L. (2022). Microbial succession and its correlation with the dynamics of volatile compounds involved in fermented minced peppers. Front. Nutr..

