# Supplementary material for: Correlation Analysis between Microbial Communities and Flavor Compounds during the Post-Ripening Fermentation of Traditional Chili Bean Paste
Source: Foods. 2024 Apr 16;13(8):1209. doi: 10.3390/foods13081209 (PMC11048965; doi:10.3390/foods13081209)
Supplement: Supplementary file 1 [file foods-13-01209-s001.zip › Supplementary Table S2.pdf]

Table S2 Bacterial and fungal alpha diversity of chili bean paste

Sample	Richness Index				Diversity Index				Coverage	
	ACE		Chao1		Simpson		Shannon		Bacteria	Fungi
	Bacteria	Fungi	Bacteria	Fungi	Bacteria	Fungi	Bacteria	Fungi		
BP0	629.27±78.54[a]	1002.05±131.67[a]	602.45±66.59[a]	1001.01±135.60[a]	0.72±0.04[a]	0.83±0.02[a]	3.15±0.24[a]	4.38±0.10[a]	0.997	0.995
BP6	518.52±45.07[b]	956.11±83.18[b]	510.81±44.39[b]	901.50±92.44[b]	0.90±0.02[a]	0.78±0.08[b]	4.70±0.33[a]	4.11±0.74[b]	0.999	0.996

Note: Different lowercase letters in the same column represent significant differences between samples ($p < 0.05$)
