# Supplementary figures and images for: Correlation Analysis between Microbial Communities and Flavor Compounds during the Post-Ripening Fermentation of Traditional Chili Bean Paste
Source: Foods. 2024 Apr 16;13(8):1209. doi: 10.3390/foods13081209 (PMC11048965; doi:10.3390/foods13081209)

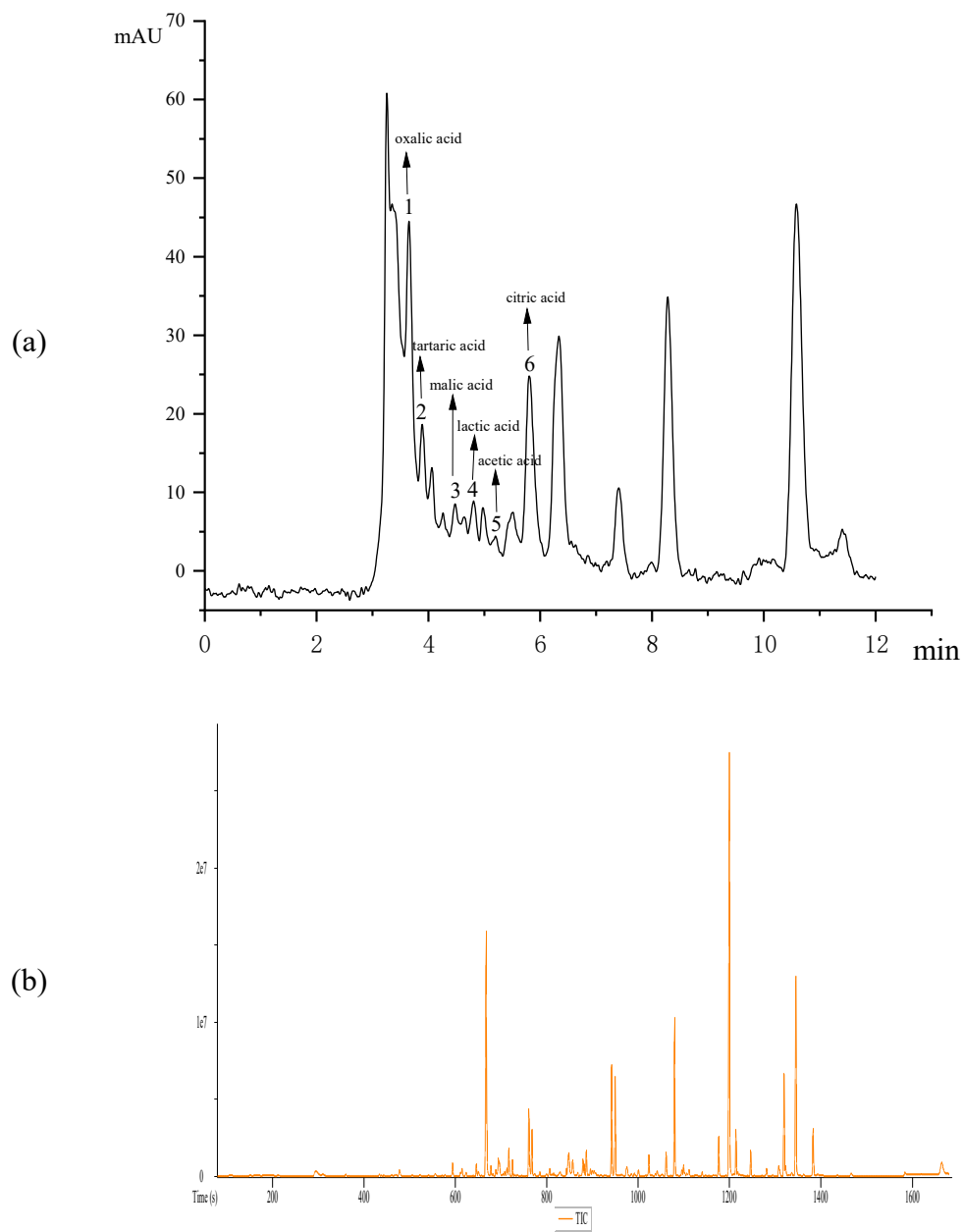

Figure S1. HPLC (a) and GC-MS (b) chromatograms of BP6

Supplement: Supplementary file 1 [file foods-13-01209-s001.zip › Supplementary FigureS1.pdf]
